# The severity of intrahepatic cholestasis during pregnancy increases risks of adverse outcomes beyond stillbirth: evidence from 15,826 patients

**DOI:** 10.1186/s12884-024-06645-2

**Published:** 2024-07-12

**Authors:** Qiulun Zhou, Yi Yuan, Yuying Wang, Zhuoqi He, Yannei Liang, Suyi Qiu, Yiting Chen, Yiru He, Zi Lv, Huishu Liu

**Affiliations:** 1 Clinical Data Center, Guangzhou Women and Children’s Medical Center, Guangzhou Medical University, Guangzhou, China; 2 School of Pediatrics, Guangzhou Medical University, Guangzhou, China; 3 Department of Obstetrics, Guangzhou Women and Children’s Medical Center, Guangzhou Medical University, Guangzhou, China

**Keywords:** Intrahepatic cholestasis during pregnancy, Adverse pregnancy outcomes, Dose-response meta-analysis

## Abstract

**Background:**

What kinds of fetal adverse outcomes beyond stillbirth directly correlate to the severity of intrahepatic cholestasis during pregnancy (ICP) remained tangled. Herein, we conducted a retrospective cohort study and a dose-response meta-analysis to speculate the association between the severity of ICP and its adverse outcomes.

**Methods:**

We retrospectively collected a cohort of ICP patients from electronic records from Guangzhou Women and Children’s Medical Center between Jan 1st, 2018, and Dec 31st, 2022. Also, we searched PubMed, Cochrane, Embase, Scopus, and Web of Science to extract prior studies for meta-analysis. The Kruskal-Wallis test, a one-way or two-way variants analysis (ANOVA), and multi-variant regression are utilized for cohort study. One stage model, restricted cubic spline analysis, and fixed-effect model are applied for dose-response meta-analysis. The data analysis was performed using the R programme.

**Results:**

Our cohort included 1,289 pregnant individuals, including 385 mild ICP cases, 601 low moderate ICP cases, 282 high moderate ICP cases, and 21 severe ICP cases. The high moderate bile acid levels were correlated to preterm birth [RR = 2.14, 95%CI 1.27 to 3.62), *P* < 0.01], and preterm premature rupture of membranes [RR = 0.34, 95%CI 0.19 to 0.62), *P* < 0.01]. We added our cases to cases reported by other studies included in the meta-analysis. There were 15,826 patients included in dose-response meta-analysis. The severity of ICP was associated with increased risks of stillbirth, spontaneous preterm birth, iatrogenic preterm birth, preterm birth, admission to neonatal intensive care unit, and meconium-stained fluid (*P* < 0.05).

**Conclusions:**

Our study shows the correlation between the severity of ICP and the ascending risks of stillbirth, preterm birth, and meconium-stained fluid, providing new threshold TBA levels.

**Prospero registration number:**

CRD42023472634.

**Supplementary Information:**

The online version contains supplementary material available at 10.1186/s12884-024-06645-2.

## Introduction

Intrahepatic cholestasis of pregnancy (ICP), is the most common liver disease specific to pregnancy. The estimated prevalence of ICP ranges from 0.2% to 25.0%, with wide variations by race, region, and seasonal variation [1]. Its prevalence is elevated in South America and Northern Europe, with the highest occurrence typically observed during the winter season [2]. In China, the incidence rate is around 1.2%, with the highest incidence rate in the Yangtze River basins [3]. ICP is a reversible disease potentially co-existing with pruritus, elevated serum aminotransferase, or abnormal total bile acid level (TBA ≥ 10µmol/L), and TBA level (10–19µmol/L as mild, 20–39µmol/L as low moderate, 40–99µmol/L as high moderate ICP, and ≥ 100µmol/L as severe) serves as the primary indicator for diagnosis and categorization of its severity.

Previous studies documented relationships with ICP and the occurrence of adverse pregnancy outcomes like spontaneous and iatrogenic preterm delivery, amniotic fluid meconium staining, and even stillbirth [4–6]. The pathophysiological mechanisms underlying the aforementioned complications may be multifactorial. First, abnormally elevated bile acid served as a vasoconstrictive agonist in the maternal-fetal interface, causing insufficient placental blood supply and chronic hypoxia, thus possibly triggering malnutrition, neonatal asphyxia, and even stillbirth [7–9]. Second, bile acid is capable of activating oxytocin receptors, and forth reinforcing the contraction of myometrium, which may be the culprit of spontaneous preterm birth [10].

While previous research has established an association between elevated TBA levels or the severity of ICP and an elevated risk of stillbirth, the impact of its severity on other adverse outcomes remains inconclusive. Herein, we collected a large population to delve into the dose-response association between the TBA levels and risks of adverse obstetric outcomes beyond stillbirth.

## Materials and methods

### Patients

The diagnosis of ICP is based on the measurement of TBA level, with a threshold of 10µmol/L. The retrospective cohort of pregnant individuals with TBA ≥ 10µmol/L was derived from Guangzhou Women and Children’s Center from January 1st, 2018 to December 31st, 2022. In our study, we excluded patients with twin or multiple pregnancies, hepatitis B, or other severe infectious diseases. Characteristics of pregnant individuals, baseline information of neonates, and adverse pregnancy outcomes were extracted from an electronic medical system. The process of data collection has been approved by the institutional review board approval of Guangzhou Women and Children’s Medical Center.

### Search strategy

In the meta-analysis section of our study, we conducted a comprehensive analysis of existing research literature. Two researchers independently searched PubMed, Scopus, Cochrane Library, and Embase databases, using MeSH terms and Entry terms, and screened eligible articles based on inclusion and exclusion criteria by October 3rd, 2023. The MeSH words include "Intrahepatic Cholestasis of Pregnancy". However, a part of the free term which had not to correspond with Mesh terms, including"Familial intrahepatic cholestasis of pregnancy", "Recurrent intrahepatic cholestasis of pregnancy", "Pregnancy-related cholestasis" and "Pregnancy-Related Cholestasis". We also checked the list of references and performed citation searching (Web of Science, v.5.23.2, and ClinicalTrials.gov up to October 3rd, 2023) of included studies to identify other potentially relevant studies.

### Included criteria and excluded criteria for meta-analysis

The inclusion criteria are as follows: (1) patients with ICP; (2) Singleton pregnancies; (3) Studies with minimal number of multiple pregnancies [11–15]; (4) Patients were grouped by TBA levels. The exclusion criteria are defined as follows: (1) patients suffering from hepatic diseases; (2) patients with other complications of pregnancy including hematological disease, acute abdomen, hyperthyroidism, and other conditions; (3) patients with fetal anomalies.

### Data extraction and quality assessment for meta-analysis

Two investigators independently conducted the data extraction procedure. When dissents occurred between the two investigators, a third-party investigator assessed the study findings.

Two researchers used the Newcastle-Ottawa Scale (NOS) to evaluate the quality of the articles. During the evaluation process, each aspect is assigned a certain number of points, with a total of 9 points. A score of 7–9 is considered high-quality research, 4–6 is moderate-quality research, and less than 4 is considered low-quality research.

### Data analysis

In our cohort, we identified the severity of ICP by pregnancy peak TBA levels (µmol/L). Further, we classified the total individuals into the following categories: mild ICP (10–19), low moderate ICP (20–39), high moderate ICP (40–99), and severe ICP (≥ 100). The cohort study employed a range of statistical methods: The Shapiro-Wilk test was utilized for assessing the normality of the data, while ANOVA and the Kruskal-Wallis test were employed for comparing differences among multiple groups. Additionally, linear regression and logistic regression were used to examine the association between TBA levels and adverse outcomes.

We calculated or extracted the 95% confidence interval (CI), the log-transformed RR, the mean number, and SDs for the “1stage” dose-response meta-analysis. Knots of restricted cubic spline (RCS) analysis were set at percentiles 5.0%, 35.0%, 65.0%, and 95.0%. Underpinned by a fixed effect model meta-analysis, we visualized the pooled relationship between different doses of TBA and adverse pregnancy outcomes via RCS. The wald test was conducted to estimate the non-linear relation of the RCS model. To assess the heterogeneity, we performed the Cochrane Q test and computed the *I*^*2*^ value. The publication bias was tested via Egger’s test. In this study, data analysis was performed by R program 4.3.0. completely. *P* value < 0.05 is the token of statistical significance.

## Results

### Patients

During the period from January 1, 2018, to December 31, 2022, there were 3,252 cases of ICP diagnosed in our hospital and 2,047 mothers had a record of delivery in the hospital, of which 1,943 were singleton pregnancies and 104 were twin pregnancies. After excluding missing data and twin pregnancies, the cohort study comprised 1,425 individuals. Subsequently, 136 patients with hepatitis B infection were excluded, resulting in a final inclusion of 1,289 cases.

**Fig. 1:**
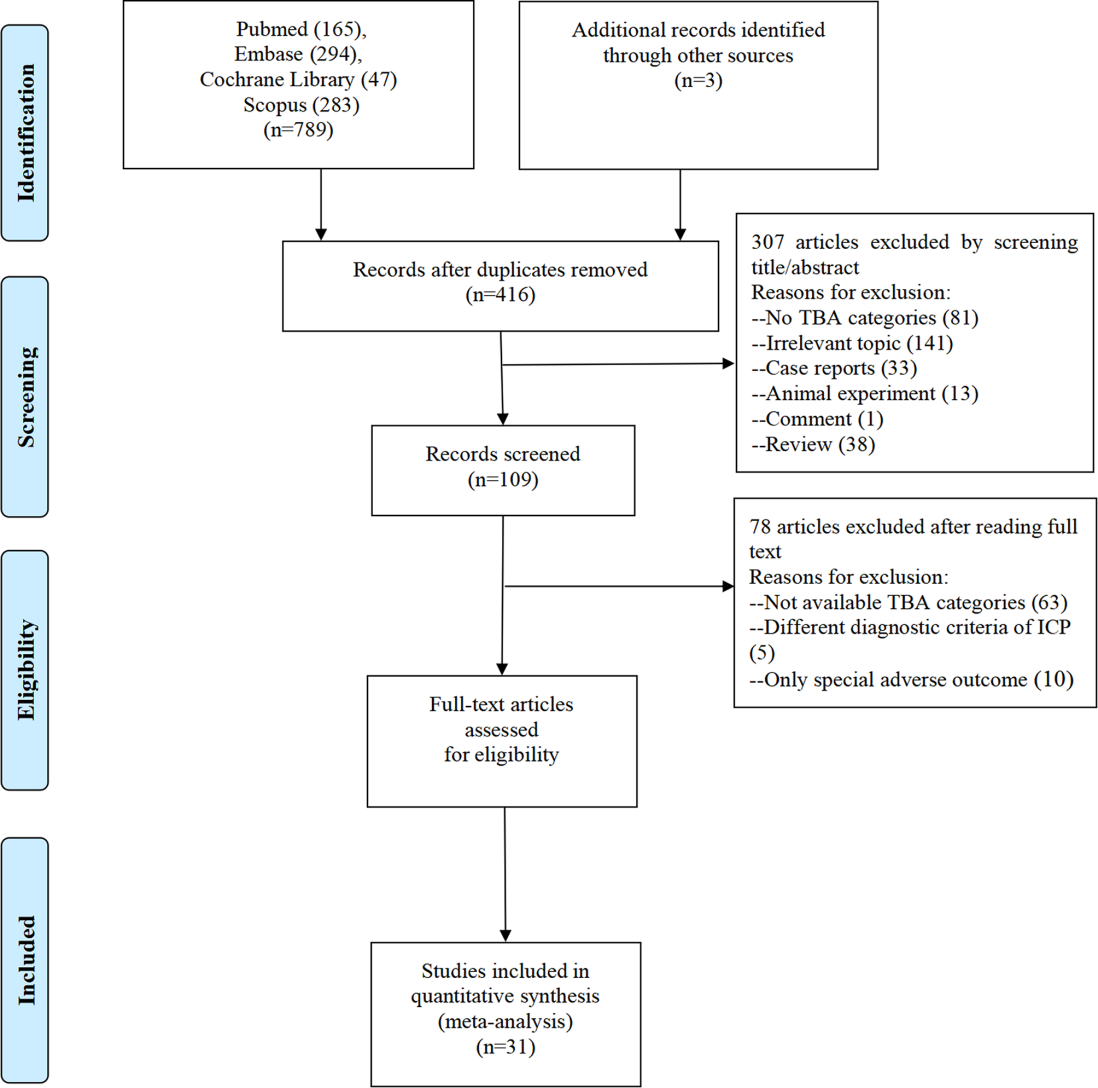
Flow graph of study selection

Figure 1 shows the results of the article research filtering. A total of 789 studies were collected from PubMed (*n* = 165), Embase (*n* = 294), Cochrane Library (*n* = 47), Scopus (*n* = 283), and additional records were identified through other sources (*n* = 3). The remaining 416 articles were found to be relevant for this meta-analysis after excluding duplications and studies that did not align with inclusion criteria. 78 articles were excluded after evaluating the full text. Eventually, 31 articles were enrolled [7, 11–40]. Additionally, we obtained the ICP cohort from the Guangzhou Women and Children’s Center and included it in the dose-response meta-analysis.

### Baseline information

In the cohort study, among these 1,289 pregnant individuals, 385 had mild ICP, 601 cases in the low moderate ICP group, 282 cases in the high moderate ICP group, and 21 cases in the severe ICP group. In these groups, there was no statistical difference between the severity of ICP and the number of parities (*P* = 0.16), gravidity (*P* = 0.93), gestational diabetes mellitus (*P* = 0.29), and the mode of delivery (*P* = 0.07) Among patients with varying degrees of ICP, differences were observed in maternal weight (*P* < 0.01), body mass index (*P* < 0.01), systolic blood pressure (*P* = 0.01), gestational week (*P* < 0.01), and report week (*P* < 0.01)(Table 1).

### Quality assessment and study characteristics

The characteristics of the eligible articles for meta-analysis (comprising a total of 32 articles, including 19 retrospective cohorts, 9 case-control cohorts, and 4 prospective cohorts) were summarized in Supplementary Table 1. Out of the thirty-two studies included in the analysis, thirty were classified as high quality based on the NOS assessment, while the remaining two studies were determined to be of moderate quality. All studies were published between 2004 and 2023. Our study included a total of 15,862 cases of ICP, with the number of cases in each study ranging from 47 to 4,329. The follow-up duration across the studies ranged from 2 years to 15 years.

**Table 1:** Baseline information of the cohort with 1,289 ICP patients

	Mild (*N* = 385)	Low Moderate (*N* = 601)	High Moderate (*N* = 282)	Severe (*N* = 21)	Overall (*N* = 1289)	*P*-value
**Age, mean ± SD**	30.5 (4.56)	30.7 (4.57)	30.8 (4.62)	31.3 (4.95)	30.7 (4.58)	0.72
**Height, mean ± SD**	159 (5.14)	159 (5.28)	159 (5.01)	158 (5.03)	159 (5.18)	0.15
**Weight, mean ± SD**	66.3 (10.2)	65.5 (8.76)	63.2 (9.54)	60.0 (6.75)	65.2 (9.46)	< 0.01
**BMI**, **median[min, max]**	25.7 [14.2, 57.8]	25.4 [17.6, 36.1]	24.9 [19.4, 39.1]	24.1 [18.0, 28.4]	25.4 [14.2, 57.8]	< 0.01
**SBP, mean ± SD**	119 (12.9)	117 (10.9)	116 (11.1)	113 (11.4)	117 (11.6)	0.01
**DBP, mean ± SD**	76.2 (8.85)	75.5 (8.69)	74.6 (8.20)	72.7 (10.0)	75.4 (8.67)	0.06
**Parity**, **median[min, max]**	0 [0, 3]	1 [0, 4]	0 [0, 3]	0 [0, 2]	0 [0, 4]	0.16
**Gravidity, median[min, max]**	2 [1, 7]	2 [1, 8]	2 [1, 7]	2 [1, 7]	2 [1, 8]	0.93
**Gestational week, median[min, max]**	38.7 [27.0, 41.1]	38.0 [31.0, 41.3]	37.7 [30.0, 41.0]	36.0 [33.0, 38.4]	38.0 [27.0, 41.3]	< 0.01
**Report week, median[min, max]**	37.0 [0, 41.0]	35.0 [0, 41.0]	36.0 [0, 41.0]	34.0 [8.00, 38.0]	36.0 [0, 41.0]	< 0.01
**GDM, n (%)**	43 (15.2%)	121 (20.1%)	60 (15.6%)	3 (14.3%)	227 (17.6%)	0.29
**Birth sex, n (%)**						0.96
boy	206 (53.5%)	336 (55.9%)	154 (54.6%)	11 (52.4%)	707 (54.8%)	
girl	179 (46.5%)	265 (44.1%)	128 (45.4%)	10 (47.6%)	582 (45.2%)	
**Delivery mode, n (%)**						0.07
Viginal	230 (59.7%)	352 (58.6%)	157 (55.7%)	6 (28.6%)	745 (57.8%)	
Cesarean	155 (40.3%)	249 (41.4%)	125 (44.3%)	15 (71.4%)	544 (42.2%)	

*BMI: body mass index; SBP: Systolic blood pressure; DBP: Diastolic blood pressure; GDM: Gestational diabetes melitus

### Primary outcomes

#### Results of cohort study

Table 2 details the major outcome indicators, including mothers and infants. The difference in preterm birth (*P* < 0.01) and PROM (*P* = 0.01) between different severity groups of ICP was statistically significant. Through univariate analysis, we observed a significant association between the severity of ICP and an elevated incidence of preterm birth (*P* < 0.01), except for the mild ICP group. Before childbirth, our data revealed that, except for the severe ICP group, increasing TBA concentrations were associated with a decreased risk of preterm premature rupture of membranes in the moderate ICP group (RR < 1, *P* ≤ 0.05). With regards to the relationship between ICP severity and fetal outcomes, ICP severity was significantly correlated with newborns’ birth height and birth weight (*P* < 0.01). However, our analysis revealed no significant correlation between ICP severity and Apgar scores.

Moreover, to account for potential confounding factors, we incorporated covariates into our analysis and employed regression model to examine the specific impact of ICP severity on outcomes within each group. Table 3 displays indicators related to motherhood after adjustment for relevant variables. After adjusting for potential confounders including report week, BMI, and SBP, it was revealed that high moderate intrahepatic cholestasis in pregnancy remained significantly associated with preterm birth [RR = 2.14, 95%CI (1.27, 3.62), *P* < 0.01], and preterm premature rupture of membranes [RR = 0.34, 95%CI (0.19, 0.62), *P* < 0.01]. When controlling for the same factors, severe ICP was associated with preterm birth [RR = 12.45, 95%CI (4.74, 32.74), *P* < 0.01]. In addition, high moderate ICP was also associated with hypertension disorder of pregnancy [RR = 0.41, 95%CI (0.20, 0.83), *P* = 0.01] after adjusted for the potential confounders of report week, gestational week and BMI. In the indicators of fetal birth weight, birth height, Apgar score in 1 min, Apgar score in 5 min, and Apgar score in 10 min, there was no statistical significance when covariates were included to adjust the regression model except for the high moderate group was adversely associated with the score of Apgar1 [RR = 0.12, 95%CI (0.02, 0.22), *P* = 0.02]. (Table 4)

**Table 2:** Adverse pregnancy outcomes of the cohort with 1,289 ICP patients

	Mild (*N* = 385)	Low Moderate (*N* = 601)	High Moderate (*N* = 282)	Severe (*N* = 21)	Overall (*N* = 1289)	*P*-value
**Preterm birth, n (%)**	27 (7.0%)	40 (6.7%)	40 (14.2%)	10 (47.6%)	117 (9.1%)	< 0.01
**Hypertension disorder of pregnancy, n (%)**	32 (8.3%)	38 (6.3%)	13 (4.6%)	1 (4.8%)	84 (6.5%)	0.43
**PROM, n (%)**	55 (14.3%)	62 (10.3%)	16 (5.7%)	3 (14.3%)	136 (10.6%)	0.01
**Meconium-stained fluid, n (%)**	18 (4.7%)	18 (3.0%)	6 (2.1%)	1 (4.8%)	43 (3.3%)	0.44
**Polyhydramnios, n (%)**	14 (3.6%)	12 (2.0%)	8 (2.8%)	0 (0%)	34 (2.6%)	0.55
**Oligoamnios, n (%)**	23 (6.0%)	19 (3.2%)	6 (2.1%)	0 (0%)	48 (3.7%)	0.07
**Birth height, mean ± SD**	49.2 (2.25)	49.0 (1.90)	48.4 (2.32)	46.3 (2.72)	48.9 (2.16)	< 0.01
**Birth weight, mean ± SD**	3110 (474)	3040 (431)	2920 (425)	2570 (395)	3030 (451)	< 0.01
**Apgar1**	9.00 [3.00, 10.0]	9.00 [5.00, 10.0]	9.00 [1.00, 10.0]	9.00 [8.00, 10.0]	9.00 [1.00, 10.0]	0.12
**Apgar5**	10.0 [6.00, 10.0]	10.0 [8.00, 10.0]	10.0 [4.00, 10.0]	10.0 [8.00, 10.0]	10.0 [4.00, 10.0]	0.09
**Apgar10**	10.0 [3.00, 10.0]	10.0 [8.00, 10.0]	10.0 [4.00, 10.0]	10.0 [8.00, 10.0]	10.0 [3.00, 10.0]	0.49

* PROM, preterm prelabor rupture of membranes

**Table 3:** Association of ICP severity and maternal outcome

	RR	95%CI	*P*-value
**Preterm birth**			
Low Moderate	0.92	(0.55, 1.54)	0.75
High Moderate	2.14	(1.27, 3.62)	< 0.01
Severe	12.45	(4.74, 32.74)	< 0.01
**Preterm premature rupture of membranes**			
Low Moderate	0.67	(0.45, 1.00)	0.05
High Moderate	0.34	(0.19, 0.62)	< 0.01
Severe	0.95	(0.27, 3.39)	0.94
**Hypertension disorder of pregnancy**			
Low Moderate	0.72	(0.43, 1.22)	0.22
High Moderate	0.41	(0.20, 0.83)	0.01
Severe	0.16	(0.02, 1.38)	0.10

*Group, report week, BMI, and SBP were adjusted for preterm birth and Preterm premature rupture of membranes; group, report week, gestational week, and BMI were adjusted for Hypertension disorder of pregnancy

**Table 4:** Association of ICP severity and fetal outcomes

	Beta	Se	95% CI	*P*-value
**Birth weight**				
Low Moderate	10.84	22.86	(-33.97, 55.64)	0.64
High Moderate	-3.93	27.97	(-58.76, 50.90)	0.89
Severe	40.37	80.52	(-117.456, 198.20)	0.62
**Birth height**				
Low Moderate	0.08	0.11	(-0.14, 0.30)	0.50
High Moderate	-0.03	0.14	(-0.30, 0.24)	0.83
Severe	-0.36	0.40	(-1.15, 0.42)	0.36
**Apgar1**				
Low Moderate	0.002	0.04	(-0.08, 0.08)	0.95
High Moderate	0.12	0.05	(0.02, 0.22)	0.02
Severe	0.25	0.15	(-0.04, 0.54)	0.09
**Apgar5**,				
Low Moderate	0.02	0.03	(-0.03, 0.07)	0.41
High Moderate	0.03	0.03	(-0.04, 0.09)	0.43
Severe	0.03	0.09	(-0.15, 0.22)	0.73
**Apgar10**				
Low Moderate	0.03	0.02	(-0.01, 0.08)	0.17
High Moderate	0.006	0.03	(-0.05, 0.06)	0.82
Severe	0.06	0.09	(-0.11, 0.22)	0.51

*All outcomes were adjusted with the group, report week, BMI, SBP, and gestational week

#### Results of meta-analysis

31 published studies and our retrospective cohort fulfilled the inclusion and exclusion criteria, and data of 15,826 patients comprising their peak TBA levels and perinatal complications were aggregated for dose-response meta-analysis.

**Fig. 2:**
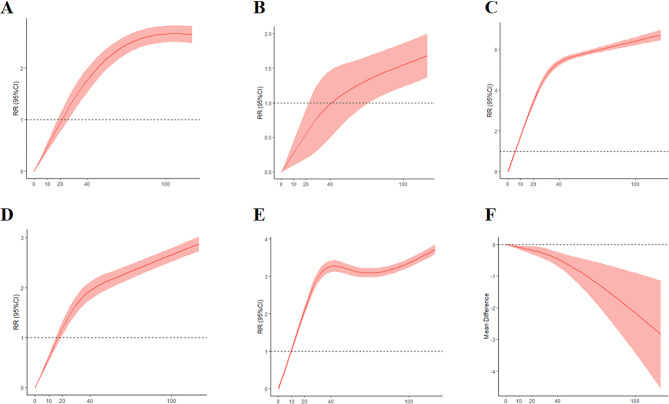
(**A**) Non-linear regression model of association between TBA levels and spontaneous preterm birth. (**B**) Non-linear regression model of association between TBA levels and iatrogenic preterm birth. (**C**) Non-linear regression model of association between TBA levels and stillbirth. (**D**) Non-linear regression model of association between TBA levels and meconium-stained fluid. (**E**) Non-linear regression model of association between TBA levels and the admission of neonatal intensive care unit. (**F**) Non-linear regression model of the association between TBA levels and birthweight

Consisting of 4,051 patients, Fig. 2A revealed TBA ≥ 20µmol/L is correlated with ascended risks of spontaneous preterm (*P* < 0.001). Figure 2B, which included 1,781 ICP patients, demonstrated a statistical relationship between TBA levels and iatrogenic preterm labor when TBA ≥ 40µmol/L (*P* < 0.001). Additionally, evidence from 8,278 patients indicated that the severity of ICP was associated with an increased incidence of stillbirth (*P* < 0.001), with a notable RR ≥ 1 observed when TBA ≥ 10µmol/L(Fig. 2C). Figure 2D disclosed a concomitant of the rising risk of meconium-stained fluid and ascending TBA levels when TBA ≥ 20µmol/L. In Fig. 2E, data from 5,610 patients revealed that elevated TBA levels were linked to the admission of neonatal care unit (*P* < 0.05). Furthermore, synthesized data from 7,267 patients in Fig. 2F demonstrated a significant decrease in mean birth weight as TBA levels increased (*P* < 0.05).

Notably, no statistical differences were found between TBA levels and the occurrence of fetal distress, neonatal respiratory distress syndrome, Apgar scores, postpartum hemorrhage, hyperbilirubinemia, cesarean delivery, and PROM. Supplementary Table 2 presented qualitative analysis results, showing a pooled RR of 1.00 with 0.0% heterogeneity for cesarean delivery in non-Asian countries, while Asian countries tended to perform a higher rate of cesarean delivery in the highest dose group (*P* < 0.01).

## Discussion

Our study first revealed the worsening of ICP is synchronized with increased risks of spontaneous preterm birth, iatrogenic preterm birth, meconium-stained fluid, NICU admission, and abnormal birth weight based on a large population including 15,826 patients. This is a large single-center cohort study and a dose-response meta-analysis aiming to elucidate the severity of ICP in relation to the adverse outcomes beyond stillbirth.

Our meta-analysis confirmed a significant association between elevated TBA levels and an increased risk of spontaneous preterm birth, with a dose-response relationship observed above a threshold of 20µmol/L. The relative risk of iatrogenic preterm surpassed 1.0 as the TBA concentration increased beyond 40µmol/L. To some extent, this finding is consistent with the ACOG committee’s recommendation for ICP patients [41]. Additionally, our dose-response analysis found when TBA levels reached ≥ 10µmol/L, the RR for stillbirth reached 1.0. This finding contrasts with a previous report that indicated no significant effects on stillbirth for the TBA equal to or less than 19µmol/L [42]. In 2019, a meta-analysis of 23 studies revealed that the risk of stillbirth only increased when TBA concentrations exceeded 100µmol/L, while the risk of stillbirth remained similar to the background risk at lower concentrations [43]. Conversely, our study found that the risk of stillbirth increased when TBA concentrations exceeded 10µmol/L through dose-response meta-analysis. Our aggregated data revealed a stillbirth rate of 1.69%(supplementary Table 3) between TBA concentrations of 10µmol/L and 40µmol/L, significantly higher than the background rate. Collectively, our findings suggest that TBA concentrations peaking over 10µmol/L may serve as a warning sign for stillbirth, and women who experience such peaks warrant necessary surveillance. There requires a larger population study to estimate the threshold TBA levels showing risks of stillbirth. Also, our findings challenge previous research by showing that the risk of meconium-stained fluid is significant when TBA levels reach 20µmol/L, rather than the previously reported threshold of 40µmol/L [44].

The clinical and research implications stem from our findings, and we reckoned that regular monitoring of TBA for its volatile nature in advanced pregnancy is essential due to the various developmental changes the fetus undergoes in utero. While our results do not directly associate the optimal timing of TBA level monitoring with specific risks, expert opinions from Puljic and Lo advised 36 weeks could be the optimum time to prevent neonatal complications [45, 46]. Prospective studies with larger populations and pilot timing will be solutions to the current challenges of ICP. Our results also identified a clear relationship between TBA concentrations and the increasing risk of meconium-stained fluid and spontaneous preterm, and importantly the TBA concentration over 20µmol/L is the threshold. More frequent monitoring of the fetus and possible neonatal intensive care should be advised to patients whose TBA is beyond the threshold.

Our study has several limitations. Firstly, despite the inclusion of 1,289 individuals in the cohort study, there is a paucity of patients suffering from severe ICP (*n* = 22), which may impact the reliability of the results for severe ICP. Secondly, the retrospective nature of the cohort study means that clinicians were not able to manage all the ICP patients under an identical therapeutic regimen. Additionally, in the meta-analysis, variations in TBA sample collection, like the sample collected at fasting and postprandial phases, could lead to the over-diagnosis or analysis inaccuracy of ICP.

## Conclusion

In conclusion, a large cohort study and meta-analysis involving 15,826 patients demonstrates a significant correlation between ICP and adverse outcomes, including preterm birth, meconium-stained amniotic fluid, and stillbirth. The study also establishes new threshold levels for TBA, providing critical insights for clinical management.

### Electronic supplementary material

Below is the link to the electronic supplementary material.

Supplementary Material 1

Supplementary Material 2

Supplementary Material 3

## Data Availability

The datasets analyzed during the current study are not publicly available due to privacy but are available from the corresponding author on reasonable request.

## Abbreviations

ICP: intrahepatic cholestasis during pregnancy
ANOVA: a one-way or two-way variants analysis
TBA: total bile acid
PE: pre-eclampsia
HBsAg: B surface antigen
PROM: pretermprelabor rupture of membranes
NICU: neonatal intensive care unit
NOS: Newcastle-Ottawa Scale
RR: relative risk
SD: standard deviation
OR: odds ratio
CI: confidence interval
RCS: restrict cubic spline
SBP: systolic blood pressure
DBP: diastolic blood pressure
GDM: gestational diabetes Mellitus
ELBW: extremely low birth weight infants
VLBW: very low birth weight infants
LBW: low birth weight infants
NBW: normal birth weight
