# Supplementary material for: The severity of intrahepatic cholestasis during pregnancy increases risks of adverse outcomes beyond stillbirth: evidence from 15,826 patients
Source: BMC Pregnancy Childbirth. 2024 Jul 12;24:476. doi: 10.1186/s12884-024-06645-2 (PMC11241884; doi:10.1186/s12884-024-06645-2)
Supplement: Supplementary file 1 — Supplementary Material 1 [file 12884_2024_6645_MOESM1_ESM.docx]

Supplementary Table 1. Basic information of included studies in meta-analysis.

| **ID** | **Author (last name)** | **Published year** | **Time range** | **Continent** | **Country** | **Study type** | **Population** | **Dose group (TBA levels)** | **NOS score** |  |
| --- | --- | --- | --- | --- | --- | --- | --- | --- | --- | --- |
| **1** | Ataalla | 2016 | Jan 2011 to March 2013 | Africa | Egypt | retrospective cohort | 98 | 25 | 8 |  |
|  |  |  |  |  |  |  |  | 55 |  |  |
| **2** | Brouwers | 2015 | Jan 2005 to Aug 2012 | European | Netherlands | case-control | 215 | 24.5 | 7 |  |
|  |  |  |  |  |  |  |  | 69.5 |  |  |
|  |  |  |  |  |  |  |  | 129.5 |  |  |
| **3** | Çelik | 2019 | 2012 to 2018 | Asia | Turkey | retrospective cohort | 370 | 25 | 8 |  |
|  |  |  |  |  |  |  |  | 55 |  |  |
| **4** | Furrer | 2016 | Nov 2004 to Nov 2014 | European | Switzerland | case-control | 345 | 25 | 5 |  |
|  |  |  |  |  |  |  |  | 69.5 |  |  |
|  |  |  |  |  |  |  |  | 129.5 |  |  |
| **5** | Garcia-flores | 2015 | Jan 2012 to May 2014 | European | Spanish | retrospective cohort | 47 | 24.5 | 8 |  |
|  |  |  |  |  |  |  |  | 54.5 |  |  |
| **6** | Glantz | 2004 | Feb 1st 1999 to Jan 31st 2002 | Europe | Sweden | prospective cohort | 505 | 24.5 | 6 |  |
|  |  |  |  |  |  |  |  | 54.5 |  |  |
| **7** | Golbasi | 2022 | Jan 2019 to Dec 2021 | Asia | Turkey | case-control | 126 | 24.5 | 6 |  |
|  |  |  |  |  |  |  |  | 54.5 |  |  |
| **8** | Gupta | 2022 | Sept 2018 to March 2020 | Asia | India | prospective cohort | 196 | 14.5 | 8 |  |
|  |  |  |  |  |  |  |  | 24.5 |  |  |
|  |  |  |  |  |  |  |  | 34.5 |  |  |
|  |  |  |  |  |  |  |  | 44.5 |  |  |
| **9** | Guszczynska-Losy | 2020 | Jan 2017 to Dec 2018 | Europe | Poland | prospective cohort | 86 | 24.5 | 9 |  |
|  |  |  |  |  |  |  |  | 54.5 |  |  |
| **10** | Herrera | 2017 | 2005 to 2015 | North America | USA | retrospective cohort | 487 | 24.5 | 7 |  |
|  |  |  |  |  |  |  |  | 69.5 |  |  |
|  |  |  |  |  |  |  |  | 114.5 |  |  |
| **11** | Huang | 2022 | Jan 2018 to Jan 2020 | Asia | China | retrospective cohort | 500 | 25 | 8 |  |
|  |  |  |  |  |  |  |  | 55 |  |  |
| **12** | Jhirwal | 2022 | Jan 1st 2019 to Jan 1st 2021 | Asia | India | retrospective cohort | 152 | 24.5 | 8 |  |
|  |  |  |  |  |  |  |  | 69.5 |  |  |
|  |  |  |  |  |  |  |  | 129.5 |  |  |
| **13** | Jin | 2014 | Feb 1st 1993 to Jan 31st 2014 | Asia | China | retrospective cohort | 371 | 25 | 8 |  |
|  |  |  |  |  |  |  |  | 55 |  |  |
| **14** | Juusela | 2019 | Feb 1st 2013 to Dec 31st 2017 | Asia | Israel | retrospective cohort | 61 | 24.5 | 8 |  |
|  |  |  |  |  |  |  |  | 69.5 |  |  |
|  |  |  |  |  |  |  |  | 129.5 |  |  |
| **15** | Kawakita | 2015 | Jan 2009 to May 2014 | North America | USA | retrospective cohort | 233 | 24.95 | 7 |  |
|  |  |  |  |  |  |  |  | 69.95 |  |  |
|  |  |  |  |  |  |  |  | 129.95 |  |  |
| **16** | Kong | 2023 | Feb 1st 2015 to Dec 31st 2019 | Asia | China | retrospective cohort | 227 | 25 | 8 |  |
|  |  |  |  |  |  |  |  | 55 |  |  |
| **17** | Li | 2023 | July 2015 to Dec 2016 | Aisa | China | case-control | 300 | 25 | 8 |  |
|  |  |  |  |  |  |  |  | 55 |  |  |
| **18** | Madazli | 2014 | Feb 2003 to Dec 2013 | Asia | Turkey | retrospective cohort | 89 | 10 | 7 |  |
|  |  |  |  |  |  |  |  | 29.5 |  |  |
|  |  |  |  |  |  |  |  | 49.5 |  |  |
| **19** | Marathe | 2017 | 2001 to 2010 | Oceania | Australia | retrospective cohort | 320 | 20 | 7 |  |
|  |  |  |  |  |  |  |  | 60 |  |  |
| **20** | Estiu | 2017 | 2009 to 2013 | South America | Argentina | prospective cohort | 382 | 14.5 | 9 |  |
|  |  |  |  |  |  |  |  | 29.5 |  |  |
|  |  |  |  |  |  |  |  | 50 |  |  |
| **21** | Mei | 2019 | Jan 2013 to May 2017 | Asia | China | retrospective cohort | 134 | 25 | 8 |  |
|  |  |  |  |  |  |  |  | 55 |  |  |
| **22** | Nezer | 2015 | Jan 2006 to June 2014 | Asia | Israel | case-control | 117 | 24.995 | 7 |  |
|  |  |  |  |  |  |  |  | 54.995 |  |  |
| **23** | Oruç | 2014 | Jan 2012 to Dec 2012 | Asia | Japan | prospective case-control | 57 | 25 | 8 |  |
|  |  |  |  |  |  |  |  | 55 |  |  |
| **24** | Premkumar | 2020 | July 1st 2017 to Nov 30 th 2018 | Asia | India | retrospective cohort | 375 | 25 | 8 |  |
|  |  |  |  |  |  |  |  | 55 |  |  |
| **25** | Proehl | 2017 | 2007 to 2014 | North America | USA | retrospective cohort | 4329 | 14.5 | 7 |  |
|  |  |  |  |  |  |  |  | 29.5 |  |  |
|  |  |  |  |  |  |  |  | 49.5 |  |  |
| **26** | Sarker | 2022 | 2005 to 2019 | North America | USA | retrospective cohort | 1202 | 14.5 | 8 |  |
|  |  |  |  |  |  |  |  | 29.5 |  |  |
|  |  |  |  |  |  |  |  | 69.5 |  |  |
|  |  |  |  |  |  |  |  | 129.5 |  |  |
| **27** | Silver | 2014 | March 2006 to Sept 2008 | North America | USA | case-control | 147 | 25 | 8 |  |
|  |  |  |  |  |  |  |  | 55 |  |  |
| **28** | Wang | 2022 | Sept 10th 2018 to June 30th 2021 | Asia | China | case-control | 52 | 24.5 | 8 |  |
|  |  |  |  |  |  |  |  | 54.5 |  |  |
| **29** | Xu | 2023 | Jan 1st 2016 to June 30th 2020 | Asia | China | retrospective cohort | 2299 | 25 | 7 |  |
|  |  |  |  |  |  |  |  | 70 |  |  |
|  |  |  |  |  |  |  |  | 130 |  |  |
| **30** | Kırlangıç | 2022 | NA | Aisa | Turkey | case-control | 60 | 24.95 | 9 |  |
|  |  |  |  |  |  |  |  | 54.95 |  |  |
| **31** | Li | 2020 | Jan 1st 2011 to Dec 31st 2014 | Asia | China | retrospective cohort | 691 | 24.95 | 8 |  |
|  |  |  |  |  |  |  |  | 54.95 |  |  |
| **32** | GWCMC_cohort | 2023 | Jan 1st 2018 to Dec 31st 2022 | Asia | China | retrospective cohort | 1289 | 10 | 9 | |
|  |  |  |  |  |  |  |  | 30 |  |  |
|  |  |  |  |  |  |  |  | 70 |  |  |
|  |  |  |  |  |  |  |  | 130 |  |  |
