# Supplementary material for: The severity of intrahepatic cholestasis during pregnancy increases risks of adverse outcomes beyond stillbirth: evidence from 15,826 patients
Source: BMC Pregnancy Childbirth. 2024 Jul 12;24:476. doi: 10.1186/s12884-024-06645-2 (PMC11241884; doi:10.1186/s12884-024-06645-2)
Supplement: Supplementary file 2 — Supplementary Material 2 [file 12884_2024_6645_MOESM2_ESM.docx]

Supplementary Table 2. Pooled RR value in the highest dose group.

| **Outcomes** | **Number of studies** | **Number of patients** | **RR** | **95% CI** | **P value** | **Heterogeneity (*I^2^* value)** |
| --- | --- | --- | --- | --- | --- | --- |
| **Spontaneous preterm birth** | 12 | 4015 | 4.72 | 4.47-4.99 | <0.01 | 95% |
| **Iatrogenic preterm birth** | 4 | 1781 | 3.00 | 2.79-3.23 | <0.01 | 97% |
| **Preterm birth** | 8 | 5847 | 4.56 | 4.43-4.70 | <0.01 | 99% |
| **Stillbirth** | 8 | 8278 | 17.31 | 16.58-18.07 | <0.01 | 99% |
| **Meconium-stained fluid** | 18 | 8021 | 4.38 | 4.23-4.54 | <0.01 | 98% |
| **Admission of neonatal intensive care unit** | 14 | 5610 | 4.37 | 4.17-4.58 | <0.01 | 98% |
| **Ceserean delivery** | 20 | 9343 | 1.38 | 1.30-1.47 | <0.01 | 84% |
| Subgroup of ceserean delivery |  |  |  |  |  |  |
| Patients from Asia | 12 | 6631 | 1.49 | 1.39-1.60 | <0.01 | 88% |
| Patients from Other continents | 8 | 2712 | 1.00 | 0.87-1.16 | 0.56 | 0% |
